# Tailoring Antibiotic Duration for Respiratory Tract Infections in Primary Care: Protocol for a Pragmatic Randomized Controlled Trial Study (STORM)

**DOI:** 10.2196/75453

**Published:** 2025-10-20

**Authors:** Rosa Morros, Ana Moragas, Ana García-Sangenís, Ramon Monfà, Marc Miravitlles, Laura Vallejo-Torres, Carmen I Jarca, Carl Llor

**Affiliations:** 1 Institut Universitari d'Investigació en Atenció Primària Jordi Gol Barcelona Spain; 2 Instituto de Salud Carlos III CIBER de Enfermedades Infecciosas Madrid Spain; 3 Universitat Autònoma de Barcelona Bellaterra Spain; 4 Jaume I Health Centre University Rovira i Virgili Tarragona Spain; 5 Pneumology Department Vall d'Hebron Hospital Universitari Barcelona Spain; 6 Department of Quantitative Methods in Economics and Management Universidad de Las Palmas de Gran Canaria Las Palmas de Gran Canaria Spain; 7 OSI Tolosaldea, Osakidetza Andoain Health Center Andoain Spain; 8 Department of Public Health University of Southern Denmark Odense Denmark

**Keywords:** respiratory tract infections, anti-bacterial agents, drug resistance, microbial, patient-centered care, deprescriptions, duration, drug-related side effects and adverse reactions, randomized clinical trial

## Abstract

**Background:**

Combating the rise of drug-resistant organisms and minimizing side effects requires a shift in how we approach the duration of antibiotic therapy. A promising strategy involves tailoring the length of antibiotic therapy to patients’ needs, allowing discontinuation once patients feel better.

**Objective:**

This study aims to assess whether shortening antibiotic therapy based on patients’ recovery time is as effective as completing the full course in treating acute respiratory tract infections (RTIs).

**Methods:**

We plan to enroll a minimum of 474 outpatients ranging from 18 to 75 years of age with clinical features of acute RTIs across Spanish health centers. Patients diagnosed with acute lower RTIs or acute rhinosinusitis, deemed by clinicians to require a β-lactam course, will be randomized to either usual care, involving a full-course antibiotic therapy based on current guidelines, or a tailored approach. In the intervention group, patients will be advised to visit the center as soon as they feel better and are afebrile for a clinical assessment and C-reactive protein rapid testing. Treatment will be discontinued if these clinical results are normal. The primary outcome will be assessment of clinical resolution at day 14, while secondary outcomes include antibiotics given and duration; adherence to the prescribed antibiotic; other therapies given; early clinical assessment at day 7; days of severe, moderate, and any symptom, complications and reattendance within the first month; drug-related adverse events; absenteeism; and health-related quality of life. All participants will be given a symptom diary, recording their symptoms each evening. Additionally, a cost-effectiveness study and qualitative studies involving clinicians and patients aimed at exploring the strategy’s pros, cons, uptake, and satisfaction levels will be carried out.

**Results:**

The final results will be published by the end of 2027.

**Conclusions:**

We will examine whether adults who present with symptoms of RTI who are treated with antibiotic courses until they feel better are as effective as longer standard courses. It is highly important that a possible reduction in the antibiotic course as soon as the patient feels better does not compromise patients’ recovery. This comprehensive approach aims to shed light on the practicality and impact of tailoring antibiotic duration in RTIs.

**Trial Registration:**

ClinicalTrials.gov NCT06581367; https://clinicaltrials.gov/study/NCT06581367

**International Registered Report Identifier (IRRID):**

PRR1-10.2196/75453

## Introduction

The judicious use of antibiotics has played a vital role in saving millions of lives and preventing serious infectious complications. However, excessive and inappropriate antibiotic use has become a major contributor to the growing global crisis of antimicrobial resistance (AMR). Research has established a strong link between antibiotic consumption and the emergence of resistant bacteria, both at the societal and individual levels [1]. Given the limited pipeline for new antibiotic development, the responsible use of existing antibiotics, particularly in primary care, is crucial to contain AMR.

Primary care accounts for approximately 80% of all antibiotic prescriptions, with respiratory tract infections (RTIs) responsible for more than half of this total worldwide [2]. Inappropriate antibiotic use for uncomplicated RTIs remains widespread. Around 50% of general practice consultations for RTIs result in an antibiotic prescription, with large variations across practices (from 20% to 80%) [2]. In Spain, more than half of patients presenting with acute rhinosinusitis, acute bronchitis, or sore throat in primary care are treated with antibiotics [3]. This is concerning, as most uncomplicated RTIs in otherwise healthy adults are viral and self-limiting, and antibiotics offer no relevant benefit.

Despite numerous initiatives to curb unnecessary antibiotic use, the impact has been modest at best. While some local successes have been documented, most antimicrobial stewardship strategies have shown only minimal reductions in antibiotic prescribing [4]. For instance, a recent systematic review found that such programs reduce outpatient antibiotic use by just 4% on average [5]. As a result, general practitioners (GPs), who are potentially the most influential professionals in tackling the issue of AMR, continue to show a consistently high rate of inappropriate and unnecessary antibiotic prescriptions.

Traditionally, antibiotics have been prescribed for fixed durations—typically 7 to 10 days—regardless of individual patient response. However, accumulating evidence suggests that shorter courses are equally effective for many bacterial RTIs and result in fewer adverse events. The concept that “shorter is better” emerged over a decade ago and has since been supported by multiple clinical trials [6]. Except for conditions like tuberculosis and specific upper respiratory infections (eg, otitis media and streptococcal pharyngitis), shorter antibiotic courses have shown equivalent efficacy. For instance, 3-day β-lactam therapy is as effective as 8-day treatment in patients with community-acquired pneumonia who improve substantially by day 3 [7,8]. Similar findings have been replicated in acute chronic obstructive pulmonary disease (COPD) exacerbations [9] and suspected bacterial rhinosinusitis [10-12]. Even early trials of penicillin in pneumococcal pneumonia supported discontinuation after symptom resolution, with only 5% relapse [13].

Despite this, many guidelines continue to recommend longer treatment durations, often unsupported by robust evidence [14], and clinicians frequently default to 7-day prescriptions [15-17].

The notion that completing a full antibiotic course prevents resistance is deeply ingrained in medical training. However, this dogma is not evidence-based. In fact, prolonged exposure to antibiotics increases the selection pressure for resistant organisms, both in the patient and the environment [18,19]. Even short courses can have long-term ecological effects, including persistent colonization by resistant bacteria and disruption of the gut microbiota. Data indicate that beyond the first few days, the additional benefit of continuing antibiotics decreases, while the risk of harm, through side effects, superinfections, and resistance, increases [20-22]. Importantly, resistance tends to emerge not at the site of infection, but among colonizing flora elsewhere in the body [23]. Over the last years, there have been an increasing number of independent bodies, scientific academies, and institutions abandoning this dogma on completing antibiotic courses [24].

A promising strategy to reduce unnecessary antibiotic exposure involves tailoring treatment duration based on individual response. Conventional stewardship programs often fail to address overtreatment in patients who genuinely need antibiotics. Encouraging shorter, evidence-based durations may be a more practical and acceptable alternative, as shown in qualitative research [25]. The optimal antibiotic duration likely varies by individual. Factors such as age, comorbidities, immune status, bacterial load, and organ function influence how patients metabolize and respond to treatment [26,27]. A one-size-fits-all approach risks under- or overtreatment. For instance, in a landmark study, 3-day treatment was as effective as 8 days in 78.5% of patients hospitalized with mild to moderate pneumonia who had improved by day 3 [7]. It would not be unreasonable to think that the percentage of patients with pneumonia who feel better after 3 days of therapy would be even higher in primary care.

When a nonbacterial or self-limiting infection is suspected, the priority should be to avoid initiating antibiotics. For patients already on fixed-duration antibiotic courses, clinicians should consider advising discontinuation once symptoms are resolved, unless the infection is deemed potentially serious [25]. In such cases, reassessment with clinical examination or point-of-care tools like C-reactive protein (CRP) testing may be warranted before stopping treatment. Interestingly, one-third of patients already stop antibiotics on their own once they feel better. Studies using electronic monitoring show poor adherence to prescribed durations in real-world settings [28]. This emphasizes the need to align prescribing practices with patient behavior and current evidence.

Reducing antibiotic use in primary care remains challenging, particularly when faced with diagnostic uncertainty or patient expectations. Yet it appears more feasible to convince physicians to shorten therapy durations than to withhold antibiotics entirely, provided there is clear, high-quality evidence supporting this approach [29]. Emerging data from randomized trials suggest that most patients recover within 3 days, making shorter courses not only safe but preferable. Current stewardship strategies have only a limited impact, and a shift toward personalized treatment durations, based on clinical improvement and patient characteristics, offers a promising path forward [30]. Ultimately, we must transition from a disease-focused model to a patient-centered approach: we are not treating RTIs, we are treating patients with RTIs. To support this shift, we propose a pragmatic randomized clinical trial aimed at evaluating the effectiveness of individualized antibiotic duration in primary care. Such research could set a new standard for antimicrobial prescribing, reducing unnecessary exposure, preserving antibiotic efficacy, and promoting truly evidence-based care.

## Methods

### Study Design

This is the protocol for a pragmatic, randomized noninferiority trial with a two-group parallel design. The unit of randomization is the patient, and the allocation ratio between the control and intervention groups is 1:1. This protocol follows the SPIRIT (Standard Protocol Items: Recommendations for Interventional Trials) 2013 statement [31].

### Objectives

The primary aim of the STORM trial is to assess whether the clinical resolution on day 14 for adults presenting with symptoms of an acute lower RTI or acute rhinosinusitis in general practice is noninferior in the intervention group (stopping the antibiotic course as soon as patients feel better and clinical assessment is within the normal range) compared with the control group (patients completing standard antibiotic courses).

Secondary objectives of this trial are as follows:

1. Evaluate the antibiotic prescribed at the index visit and the planned and actual duration in the 2 groups.
2. Evaluate the adherence to the prescribed antibiotic in the 2 groups.
3. Evaluate the early clinical resolution on day 7.
4. Evaluate the duration of symptoms in the 2 groups: (1) duration of severe symptoms, (2) duration of moderate symptoms, and (3) total resolution of symptoms.
5. Evaluate the use of antibiotics other than the study medication and other symptomatic therapies within the first 4 weeks in the 2 groups.
6. Determine the number of reattendances to any doctor regarding the infection within the first 4 weeks in the 2 groups.
7. Determine the number of complications related to the infection within the first 4 weeks, such as visits to emergency departments, hospital admissions, in the 2 groups.
8. Determine the number of adverse events in the 2 groups.
9. Estimate the incremental cost per patient achieving clinical resolution (within-trial cost-effectiveness analysis) and the incremental cost per quality-adjusted life year (long-term cost-effectiveness analysis) in the 2 groups.
10. Determine the number of days of work absenteeism due to the infectious disease in the 2 groups.
11. Determine the improvement in health-related quality of life in the 2 groups.
12. Assess the percentage of patients assigned to the intervention group who feel better and who comply with the criteria for stopping the antibiotic therapy in the 2 groups.

We hypothesize that patients with ages ranging from 18 to 75 years who are allocated to the intervention group and are asked to discontinue antibiotic therapy as soon as they feel better and afebrile, their clinical stability criteria are normal (systolic blood pressure >90 mm Hg, axillary temperature <37 °C, respiratory rate <20 breaths/minute, and oxygen saturation >92%) and CRP falls within normal range will have a similar overall clinical efficacy at day 14 and will not present a higher reconsultation rate compared with those allocated to the control group. We expect a lower number of drug-associated adverse events in the intervention group.

### Eligibility Criteria

We will prospectively and consecutively recruit adult patients aged 18 to 75 years who present either with acute cough as the predominant symptom—considered by the GP to be a bacterial acute lower RTI, as defined by the European Society for Clinical Microbiology and Infectious Diseases [32]—or with clinically suspected acute bacterial rhinosinusitis, as defined by the American Academy of Otolaryngology–Head and Neck Surgery Foundation [33], in cases where the GP considers a standard β-lactam course to be necessary. Patients who meet criteria for hospital referral, have a known allergy to β-lactams, are immunocompromised, or are in a severe clinical condition will be excluded from the study. A detailed summary of all inclusion and exclusion criteria is provided in Textbox 1. All patients who agree to participate will provide a signed informed consent and will be informed about the nature of this study. The participating GPs will prescribe an antibiotic, and the patient will be later randomized. Patients in the intervention group will be asked to return to the center as soon as they feel better and are afebrile for an assessment of the clinical stability criteria and CRP measurement.

**Eligibility criteria.**

**Inclusion criteria (all the criteria must be met):**

- Age ranging from 18 to 75 years.
- Either acute lower respiratory tract infection (RTI) or bacterial rhinosinusitis.
- Acute lower RTI: acute cough (new or worsening of a previous cough present for 21 days or less) as the predominant symptom, accompanied by at least one other lower respiratory tract symptom—such as sputum production, dyspnea, wheezing, and chest discomfort/pain—with no alternative explanation. It is presumed to have a bacterial cause, such as community-acquired pneumonia, acute bronchitis, or acute exacerbations of chronic obstructive pulmonary disease (COPD), according to the clinician’s judgment.
- Acute bacterial rhinosinusitis: presence of purulent nasal discharge or facial pain/pressure lasting less than 4 weeks, which the clinician suspects to be bacterial due to lack of improvement within 10 days, severe symptoms from day 3, or worsening after day 5.
- General practitioners deem β-lactam therapy for at least 7 days is necessary.

**Exclusion criteria (any of the following exclusion criteria):**

- RTIs different from a lower RTI or acute rhinosinusitis.
- The doctor decides not to give an antibiotic for this RTI.
- Patients with suspected septicemia (quick sequential organ failure assessment: respiratory rate ≥22 breaths/minute, systolic blood pressure <100 mm Hg, or altered mental status with a Glasgow score < 5) ≥2.
- Patients with pneumonia and a CRB-65 (confusion, respiratory rate ≥30 breaths/minute, blood pressure < 90/60 mm Hg, or age >65 years) ≥1.
- Patients with very severe COPD (forced expiratory volume in 1 second [FEV_1_]<30%).
- Patients with COPD who have taken 4 or more antibiotic courses in the previous year, the presence of significant bronchiectasis, and isolation of Pseudomonas in a previous sputum culture.
- Patients with reported allergy to β-lactams.
- Patients who have taken antibiotics in the previous 2 weeks.
- A working diagnosis of a noninfective disorder, such as heart failure, pulmonary embolus, or esophageal reflux.
- Immunocompromised patients, such as those with HIV infection, patients taking immunosuppressive treatment, antineoplastic therapy, or systemic corticosteroids.
- Currently participating in another clinical trial.
- Previously participated in the STORM study.
- Active neoplasia.
- Terminal illness.
- Institutionalized patient.
- Inability/unable to understand or take part in the clinical trial.

GPs will be informed about managing acute RTIs with β-lactam therapy, following the specific RTIs outlined in this study and the latest Spanish primary care guidelines [34,35] (Table 1). The guidelines recommend the use of 7-day antibiotic durations. Doctors participating in this study will be provided with a summary of the current local guidelines, including indications for when to treat. However, the final decision on antibiotic prescriptions will be at the discretion of the GP. The trial is pragmatic, aiming to mirror the real-life practices of doctors in Spain. According to a recent study conducted in Barcelona in 2023, approximately 80% of all prescriptions for amoxicillin and amoxicillin-clavulanate in primary care corresponded to box sizes of 30 pills, representing a 10-day duration, while the remainder were 20-pill boxes [36]. Considering that this clinical trial is pragmatic, we will consider a minimal duration of 7 days.

**Table 1:** Guidelines for prescribing antibiotics for respiratory tract infections in primary care in non-β-lactam-allergic patients in Spain, based on the updated recommendation [34,35] (to be performed by the general practitioner).

Diagnosis	When is it appropriate to prescribe antibiotics?	First choice antibiotic
Acute rhinosinusitis^a^	Do not administer, unless there are signs and symptoms that do not improve within 10 days, severe symptoms from day 3, or worsening of symptoms from day 5	Amoxicillin and clavulanate 875-125 mg/8 h/7 d or penicillin V 1000 mg/8 h/7 d
Lower respiratory tract infection (non-COPD^b^)^c^	In acute bronchitis, antibiotics should not be given, except in specific situations. Always treat in pneumonia	Amoxicillin 1000 mg/8 h/7 d or penicillin V 1.000 mg/8 h/7 d. Amoxicillin and clavulanate 875-125 mg/8 h/7 d if age >65 years
COPD exacerbation^d^	Only if there is sputum purulence and in all very severe or very severe COPD exacerbations	Amoxicillin and clavulanate 875-125 mg/8 h/7 d

^a^Refer to the hospital if persistent temperature >39 °C, periorbital oedema, intense headache, neurological focal signs, signs of meningeal irritation, impaired consciousness, suspicion of orbital infection, or diplopia.

^b^COPD: chronic obstructive pulmonary disease.

^c^Refer to the hospital if oxygen saturation <92%, significant alterations in chest x-ray, difficulties in complying with treatment at home, decompensation of underlying illness, lack of tolerance to oral treatment, immunosuppression, or CRB65 ≥1. Refer to the hospital if CRB65 >1 (confusion, respiratory rate ≥30 breaths/min, systolic blood pressure <90 mm Hg or diastolic blood pressure <60 mm Hg, age >65 years.

^d^Impaired consciousness, cyanosis, respiratory rate ≥30 breaths/min, severe dyspnea, oxygen therapy, inability to stay at home, or oxygen saturation <92%. Patients with COPD with forced expiratory volume in 1 second (FEV_1_) <30% are excluded from this study.

Eligible participants will be informed about the STORM trial and provided with an information leaflet and a consent form from the GP. The GP will ensure that the patient understands the purpose of the project, potential benefits, risks, and procedures involved. The GP will emphasize that participation is voluntary and that the patient can decline to participate or withdraw from the project at any time without consequences. The patient is informed that consent to participate will give the primary investigator, sponsor, and controlling authorities access to obtain information in the patient’s medical records, including the electronic records, to obtain information about the patient’s health conditions. The GP will check patients against the eligibility criteria stated above and invite patients to participate if they fulfill all the inclusion criteria and none of the exclusion criteria. Patients who agree to participate will be asked to provide written consent, which will be obtained by the GP. Informed consent will be obtained prior to the collection of participant data. Participants will be informed about the storage and use of their data.

### Interventions

Patients assigned to usual care will be given a full course of the first-line antibiotic course as recommended by the updated local guidelines [34,35]: amoxicillin 750 mg 3 times daily (t.i.d.) for 7 days for acute rhinosinusitis, 1000 mg t.i.d. for 7 days for suspected pneumonia (with clavulanate if the patient is older than 65 years), and amoxicillin/clavulanate 875 mg/125 mg t.i.d. for 7 days for suspected acute exacerbations of COPD (Table 1). Nonetheless, this guideline is designed to serve as a reference for making informed and improved choices in prescribing. The decision to prescribe an antibiotic is solely at the discretion of the participating doctor. The trial is pragmatic, thus mirroring the real-life practices of doctors in Spain. Doctors may also prescribe antibiotics for longer regimens, such as 10-day durations, as shown in recent studies [36]. The decision to prescribe an antibiotic will always be made before randomizing the patient.

Patients in the intervention group will be treated accordingly, following the same recommendations as patients assigned to the control group, but they will be asked to return as soon as they feel better and are afebrile.

When deciding whether to discontinue the antibiotic course after a patient feels better during a consultation, the participating professionals must assess three key areas:

1. Overall improvement in the patient’s symptoms.
2. Evaluate the vital signs, confirming that most of them are within the normal range and that the following clinical criteria are met: temperature <37 °C, blood pressure >90/60 mm Hg, pulse <100 beats/min, respiratory rate <20 breaths/min, and oxygen saturation >92%.
3. Assess the CRP value and interpret the results according to the provided table based on current recommendations [37].

Patients who meet all these criteria will be asked to discontinue treatment. Otherwise, patients will be instructed to continue the course for 2 more days and, if they continue feeling better, to return for reassessment. In case of worsening symptoms, they will be advised to contact their doctor for clinical assessment. The doctor will then decide whether to recommend completing the full antibiotic course, changing the therapy, or making a referral.

### CRP Point-of-Care Testing Training Program

An on-the-spot one-hour training workshop in the different centers will take place before the inception of the study, with explanation of the clinical guidelines and how to perform CRP rapid testing. Participating professionals will be informed about the updated recommendations on how to interpret the CRP values and the evidence-based management, and this interpretation will depend on the day the patient feels better [37-39] (Table 2). The tests will be carried out with Affinion 2 CRP test (Abbott Diagnostics, United States). Professionals will practice using the device during a run-in period, 2 weeks before the study starts.

**Table 2:** C-reactive protein (CRP) rapid testing value interpretation [37-39].

CRP value	Observations	Decision to stop the antibiotic course
<40 mg/L	Self-limiting infection, significant improvement, or cure	It is safe to stop the course of antibiotics.
40-100 mg/L	Consider other risk factors, age, signs, comorbidity, and symptoms of the infection	It is safe to stop the course of antibiotics if the patient returns on or before day 4. In general, it is safe to stop antibiotics on day 5 or later, unless the patient has significant comorbidities or is of advanced age. Do not stop the course in patients with COPD^a^.
>100 mg/L	Reassess the patient. The doctor should reexamine the patient with RTI^b^	Do not stop the course of antibiotics.

^a^COPD: chronic obstructive pulmonary disease.

^b^RTI: Respiratory tract infection.

### Criteria for Discontinuing or Modifying Allocated Interventions

Due to the short intervention period after index consultation, the allocated intervention may only be discontinued for a given trial participant by the GP due to an unexpected event, hindering the GP from conducting or completing the allocated intervention, for example, acute worsening of the participant’s condition during index consultation, after consent has been given and allocation has been revealed. Moreover, the study may be discontinued for a given trial participant upon participant request or by withdrawal of informed consent. The data collected before the discontinuation or withdrawal of consent will be retained and used in the analyses; however, no further data will be obtained from the participant.

### Strategies to Improve Adherence to Interventions

Monthly meetings with the coordinators of the different centers will be carried out during the performance of the randomized clinical trial. Each center will have a centralized mobile phone for the study, through which patients will contact when they feel well to be directly scheduled at the center as soon as possible to assess the discontinuation of the antibiotic.

### Relevant Concomitant Care Permitted or Prohibited During the Trial

Treatments taken on a chronic basis will be permitted, such as inhaled corticosteroids, beta-agonists, anticholinergics, symptomatic treatments, and medications for other concomitant diagnoses, as all these treatments must be distributed evenly between the 2 groups. Patients taking immunosuppressive treatment, antineoplastic therapy, systemic corticosteroids, or antibiotics in the previous 2 weeks will be excluded.

### Outcomes

#### Primary Efficacy Endpoint

The primary outcome is the percentage of patients achieving clinical resolution, defined as the disappearance of fever and either the disappearance or improvement in overall condition, such that no additional antimicrobial treatment is necessary. Any other clinical outcome that does not meet the definition is considered treatment failure. The evaluation will take place on day 14 via a phone call and will always be conducted by a doctor from the team of physicians participating in the study.

#### Secondary Efficacy Endpoints

##### Outcomes Collected From the RTI Symptom Diaries

Participants will use a validated self-reported RTI symptom diary, used in previous studies by the research team [40]. Each symptom will be graded on a 7-point Likert scale (0=not affected; 1=very little problem; 2=slight problem; 3=moderately bad; 4=bad; 5=very bad; 6=as bad as it could be). Symptoms recorded include cough, phlegm, shortness of breath, wheeze, blocked/runny nose, facial pain, chest pain, sensation of fever (high temperature), muscle aching, headache, disturbed sleep, feeling generally unwell, and interference with normal activities/work. Participants will rate symptoms for 14 days and return the diary upon completion (electronically or paper-based). It will document daily antibiotic doses and other therapies. For patients who do not return diaries, a short form asking about how many days antibiotics were actually taken, whether antibiotics have been taken after being discontinued by their doctors, and details of the duration and severity of symptoms will be completed by a telephone call to the patient at day 14.

1. Duration of antibiotic therapy, in days, defined as the number of days until discontinuing treatment in both groups (mean/median)
2. Use of antibiotics other than the study medication and other symptomatic therapies within the first 2 weeks (mean/median)
3. Early clinical assessment based on the overall score at Day 7, defined as the total symptom score reported by the patient on that day (mean/median)
4. Duration of severe symptoms, in days, defined as the number of days until the last day the patient scores 5 in any of the symptoms (mean/median)
5. Duration of moderate symptoms, in days, defined as the number of days until the last day the patient scores 3 in any of the symptoms (mean/median)
6. Total resolution of symptoms, in days, defined as the number of days until the last day the patient scores 0 in all the symptoms (mean/median)
7. The duration of antibiotic therapy, in days, defined as the number of patients discontinuing treatment in both groups, and day they discontinue will also be checked via electronic medical records

Outcomes 1 to 7 from the RTI symptom diary will be calculated for each participant every day from day 0 until day 14 or when the participant has scored 0 for every item, whichever comes first.

##### Outcomes Collected From Electronic Case Report Forms, When Available, and From Follow-Up Phone Calls, Which Will Be Cross-Checked With the Patients’ Electronic Clinical Records

1. Type of antibiotic prescribed and the planned duration.
2. Number of reattendances to any doctor for new or worsening symptoms regarding the infection, within 28 days after the index consultation.
3. Number of complications related to the infection within 28 days after the index consultation, such as visits to emergency departments or hospital admissions regarding the RTI.
4. Number of days of work absenteeism due to the infectious disease, within 28 days after the index consultation.
5. Difference in health-related quality of life on days 14 and 28, compared with baseline, measured using the 5-level EQ-5D version-quality of Life instrument (ie, EQ-5D-5L) [41].
6. Drug-associated adverse events related to the antibiotic, defined as any harmful and unintended health incident—such as a symptom, an abnormal laboratory finding, or worsening of a pre-existing condition—temporally associated with the use of the antibiotic, regardless of whether it is causally related to the treatment, and antibiotics changed within the first 14 days.

##### Other Outcomes

Percentage of patients assigned to the intervention group who feel better and who comply with the criteria for stopping the antibiotic therapy, based on the information collected during the visit to the center

#### Participant Timeline

**Figure 1:**
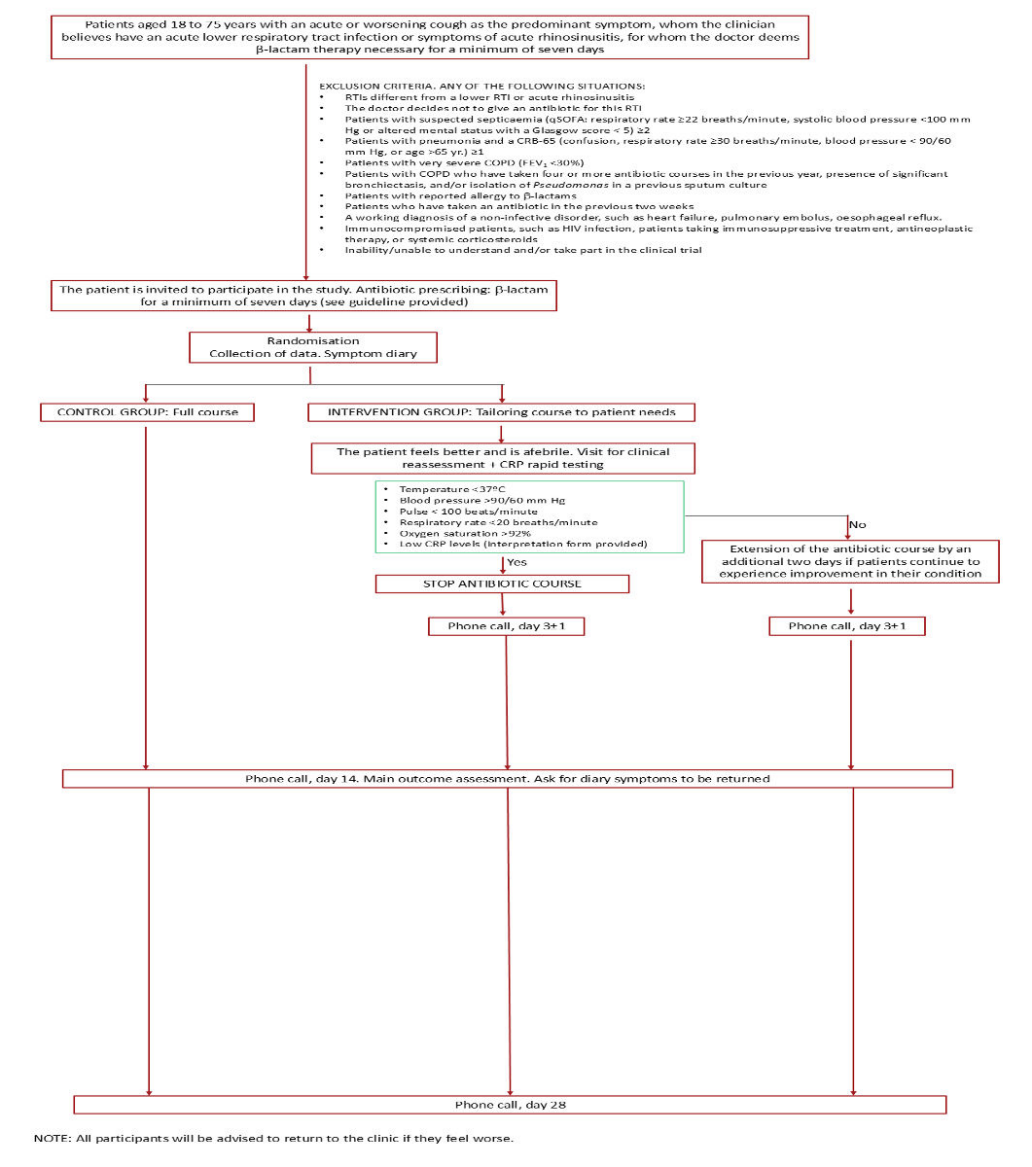
Study flowchart of the clinical trial. COPD: chronic obstructive pulmonary disease; CRP: C-reactive protein; RTI: respiratory tract infection; SOFA: sequential organ failure assessment.

A flow chart of patients is described in Figure 1. To ensure a standardized and uniform inclusion process, the principal investigator and a senior project manager will inform all participating health care professionals on the inclusion procedures for the study. Eligible participants will be identified as they present to general practice. Once the GP identifies the patient as eligible for the study and a first-line antibiotic with a -lactam is warranted, all the information about the study will be provided, and informed consent will be signed. Details of the ascertainment of visits are provided in Table 3.

**Table 3:** Ascertainment of visits for the clinical trial.

	V1^a^	V2^a^	V3^a^	V4^a^	V5^a^	V6^a^
	Face-to-face visit		Phone call			
	Day 0 (baseline visit)	From day 1^b^ (interv)	Day 3 (±1 day)	Day 7	Day 14	Day 28
Medical history	✓	✓				
Standard physical examination	✓	✓				
Eligibility criteria	✓					
Informed consent	✓					
Prescription of the antibiotic	✓					
Randomization	✓					
EQ-5D-5L questionnaire	✓				✓	✓
Hand-out of the symptom diary	✓					
CRP^c^ rapid testing		✓				
Decision to stop antibiotic course		✓				
Collection of the symptom diary		✓			✓	
Assessment of the clinical efficacy			✓	✓	✓	
Assessment of the drug adherence		✓	✓	✓	✓	
Monitoring of adverse events			✓	✓	✓	✓
Complications, re-attendance			✓	✓	✓	✓

^a^Visits 1-6.

^b^As soon as the patient feels better, only for patients in the intervention group.

^c^CRP: C-reactive protein.

Patient data will be collected at 4 specific time points for patients assigned to the control group and 5 for those allocated to the intervention:

- Day 0 (inclusion date at the center): Patients meeting the inclusion criteria will be invited to join the study by their GP. After obtaining informed consent, patients will be randomized into either the control group (receiving a full course of β-lactam therapy for at least 7 days) or the intervention group (instructed to return for assessment upon feeling better and being afebrile). The GPs will complete a case report form on day 0 (inclusion day), recording details such as age, inclusion and exclusion criteria, symptoms, date of symptom onset, comorbidities, concomitant medication, quality-of-life questionnaire (EQ-5D-5L instrument), randomization, assigned group, and details of the antibiotic administered. Patients will also be provided with a symptom diary (web-based or paper-based)
- From day 1 until the last day of the antibiotic regimen duration (at the center, only for patients assigned to the intervention group): For patients assigned to the intervention, they will be asked to return to the center for clinical reassessment and CRP rapid testing by the professional. Each center will organize ways to make it easy for patients to reach out and schedule an appointment as soon as they feel better (eg, phone number and web-based page). To decide whether to discontinue the antibiotic course when the patient reports feeling better during the consultation, three types of assessment are needed: (1) overall improvement in the patient’s symptoms; (2) signal signs are in normal range (temperature <37 °C; blood pressure >90/60 mm Hg; pulse < 100 beats/minute; respiratory rate <20 breaths/minute, and oxygen saturation >92%), and (3) CRP value is lower than the prespecified values depending on the day of the visit. All this information will be recorded in the electronic case report form (e-CRF; Table 1)
- Day 3±1 (phone call, only for patients assigned to the intervention group): A reminder phone call for all patients assigned to the intervention group will be made on this day. They will be asked how they feel and reminded to reach out to the center as soon as they feel better for a discontinuation assessment
- Day 7 (phone call): An early clinical efficacy outcome will be assessed during this call, including the clinical progression of signs and symptoms, treatment adherence, adverse reactions, any complications, reattendance visits, changes to the assigned treatment or concomitant medication, and the use of antibiotics other than the study drug
- Day 14 (phone call): The main clinical efficacy outcome and some secondary outcomes, including clinical progression of signs and symptoms, compliance with the study treatment, EQ-5D-5L instrument, adverse reactions, any complications, reattendance visits, changes in the assigned treatment or concomitant medication, including the use of antibiotics other than the study medication, will be assessed. Patients will be asked to return their diaries
- Day 28 (phone call): Patients will be contacted for information on the number of reconsultations, complications, and the quality-of-life (EQ-5D-5L) instrument

#### Sample Size

We assumed 85% clinical recovery in the group assigned to the full antibiotic course based on some studies [12,42-44]. Considering a power of 80%, a level of significance of 5%, one-sided noninferiority margin of 10%, as recommended by the guideline on the evaluation of medicinal products indicated for treatment of bacterial infections issued by the European Medicines Agency in 2022 [45], which gave a sample size of 402 patients. Assuming that the primary outcome could not be evaluated in 15% of patients, 474 patients needed to be included in the study (237 in the control group and 237 in the intervention group).

#### Recruitment

A total of 474 eligible patients will be enrolled by doctors in 25 primary care centers throughout Spain. Centers with a minimum of 3 health care professionals willing to participate in the trial will be invited to join the study. The study is planned to run from October 2025 to April 2027, including 2 winter periods (last patient recruited in March 2027). A tight follow-up on the process and different strategies will be implemented, and if needed (1) other professionals in the existing centers will be invited to participate in the study; (2) we can extend the recruitment period by an additional 2 months, until May 2027 (last follow-up in June 2027); or (3) other sites will be opened to increase the candidates to enter the study. Monthly meetings will be held with the coordinators at the different centers to maintain recruitment and enrollment activity during the inclusion period.

#### Assignment of Interventions: Allocation

##### Sequence Generation

Patients fulfilling the inclusion criteria will be asked to participate in the trial. All patients who agree to participate and provide signed informed consent will receive an increasing sequential patient number and be randomly assigned to one of the 2 treatment groups in a 1:1 ratio stratified per main diagnosis, one for acute rhinosinusitis and another one for lower RTI.

##### Concealment Mechanism

Since this study is open-label to patients and investigators, randomization will be based on investigator-blinded blocks of randomly varying size to protect against the potential predictability of treatment assignments. Blocks will be small to decrease the potential for mid-block inequality.

##### Implementation

The allocation sequences will be prepared by one remote independent researcher who is available to generate more allocation sequences.

#### Assignment of Interventions: Blinding

Owing to the type of intervention, participants and GPs are not blinded to group allocation. It is also difficult for the principal investigator and members of the research team involved in obtaining and analyzing the data (outcome assessors and data analysts) to be blinded to group allocation, as patients allocated to the intervention group have more visits scheduled.

### Data Collection and Management

#### Plans for Assessment and Collection of Outcomes

All the data is collected via e-CRFs and surveys through Research Electronic Data Capture (REDCap; Vanderbilt University), which is a secure web-based data collection platform for building and managing web-based databases and surveys [46].

#### Collection of Baseline Characteristics of GPs

The baseline characteristics of the participating GPs are collected through email correspondence and registered in REDCap by the principal investigator. The following information will be obtained: age, sex, region of Spain, type of primary health clinic, seniority as a GP, experience with randomized clinical trials, experience with research on RTIs, comfort with stopping antibiotic courses, and baseline antibiotic prescribing.

#### Collection of the Participant Data

We will collect health and personal data from the patients included in the study via REDCap, where the researchers will register basal data and evolution of the illness, and via a patient diary completed by the patients themselves. Regarding the patient’s diary, we will also create a web-based symptoms diary to make it available for all patients recruited. All patients will be able to choose between the web-based and paper version of the symptom diary. Patients will be informed about the type of data that will be collected and about their rights when being informed about the study, and will sign a consent form consenting to the use of their data. No data will be collected before the patient signs the consent form. As soon as the patient has signed the consent form, they will be given a study code and will be identified by the study code in all the study materials. The relationship between his identity and the study code will be kept in the health care center and will only be available to those who need it to fulfill their duties. Data collected in this pragmatic study is considered codified data. IDIAPJGol has license to use REDCap and the data is collected and held in IDIAPJGol servers, which comply with the European and national requirements. Data controller will be the institution (IDIAPJGol) for the study-related data and the health care institution for the health data collected to manage the patient in clinical practice. There will not be any transfer of data. Only the investigator team will have access to study datasets, and always with codified information to preserve the confidentiality of the participants involved. All the recruiting investigators will have access to REDCap platform to collect the data, each of them with their own specific credentials, and they will only have access to the patients recruited in their own setting. We will follow the FAIR (Findable, Accessible, Interoperable, and Reusable) principles: all datasets and publications will be identifiable by using a “Digital Object Identifier (DOI)” and we will share the data (as far as possible) in the Zenodo platform. We will publish the results in Open Access journals, and wherever possible, the data will be shared without delay following the Creative Commons License with Attribution (CC-BY) or the “No Rights Reserved” (CC0). Data will be saved and shared using standardized formats (txt, csv, and pdf).

### Ethical Considerations

The protocol has been approved by the Spanish Agency of Drugs and Health Products and Hospital de Sant Pau Independent Ethics Committee, Barcelona (reference number 25/233). We will follow the legislation on data protection (EU 2016/679) and its national implementation (LOPD 3/2018 of 5 Dec on the protection of personal data and guarantee of digital rights).

### Plans to Promote Participant Retention and Complete Follow-Up

The loss of follow-up data obtained from medical records and shared medication records is expected to be minimal. Outcome data from medical records be collected for all participants, except those who are discontinued by the GP or who have withdrawn informed consent before day 28. We expect some participants to be lost to follow-up in the RTI symptom diary, as we anticipate that some participants will fail to complete the electronic diary or will fail to submit the paper version to the GP. For those who do not return the diaries, participants will be contacted by phone on day 14 to gather information on antibiotic use, adherence, and symptom duration details.

### Statistical Methods

Statistical methods for primary and secondary outcomes: The characteristics of the study population will be described using frequencies for categorical variables and mean and SD for quantitative variables. To compare the clinical efficacy between the 2 strategies, we will analyze the difference in the proportion of recovered patients in each arm on day 14. Efficacy evaluation will be primarily based on intention-to-treat analysis in such a way that any event in any patient will be included in the group to which the patient was randomized, and per protocol analysis will be used as a secondary analysis. For all analyses, the level of significance will be *P*<.05.

- Interim analyses: No interim analysis is planned.
- Methods for additional analyses (eg, subgroup analyses): We will conduct subgroup analyses of the primary outcome of participants with lower RTIs, and for acute rhinosinusitis.

### Cost-Effectiveness Analyses

We will estimate the cost-effectiveness of tailoring antibiotic duration therapy for the “within-trial” period (28 days) and over the expected lifetime of patients (lifetime/long-run model).

In the within-trial analysis, cost-effectiveness will be measured by the incremental cost per patient achieving clinical resolution. In this analysis, effectiveness will be calculated using the primary endpoint of the study. Costs considered will include antibiotic prescription costs according to the duration of treatment, other drug costs, reconsultations in primary and secondary care (including hospitalizations), and costs associated with adverse events. Data on health care resource use and duration will be taken from the patients’ diaries, from the collected information in the e-CRF, and at the follow-up phone calls at days 14 and 28, and from a review of patients’ medical notes. Unit cost data will be taken primarily from national sources published by the Spanish Ministry of Health. The incremental cost-effectiveness ratio will be calculated by dividing the estimated difference in costs by the estimated differences, in effects observed between the intervention and control groups. Nonparametric methods to calculate confidence intervals around the incremental cost-effectiveness ratio based on bootstrapped estimates of the mean cost and effect differences will be used.

The intervention under evaluation in this study is likely to have an impact beyond the trial period. To capture these potential effects, we will extrapolate the results to an extended time horizon, that is, considering the remaining life expectancy of the patients. In this analysis, we will complement data from the trial with relevant data from the published literature to estimate the incremental cost per quality-adjusted life year (QALY) gained. Health-related quality of life data collected during the trial using the EQ-5D-5L questionnaire at baseline, at 14 and 28 days will be used as inputs to estimate baseline QALY values for this patient group. A decision-analytical model synthesizing all relevant information will be used to estimate the differences in lifetime QALYs and lifetime health care costs of a cohort of patients under the tailored duration strategy versus the standard of care strategy.

### Methods in Analysis to Handle Protocol Nonadherence and Any Statistical Methods to Handle Missing Data

Analyses will be performed as intention to treat (pragmatic trial) and also per protocol. The primary analysis population will comprise all participants, irrespective of follow-up.

Data will be examined for missing values and outliers. Measures of central tendency and dispersion for continuous study parameters will be portrayed. Extreme or unexpected values will be examined individually for authenticity, and data discrepancies addressed where appropriate.

### Plans to Give Access to the Full Protocol, Participant-Level Data, and Statistical Code

The full protocol, statistical code, and e-CRF templates designed for the study will be available upon request once the report has been published. Participant-level data will not be available.

## Results

Recruitment of patients will start in October 2025 and will be stopped in April 2027. The current protocol is version 3 of July 31, 2025.

## Discussion

### Principal Findings

The limited to moderate impact in reducing unnecessary antibiotic use in primary care observed in antimicrobial stewardship programs necessitates the exploration of new strategies aimed at reducing patients’ exposure to unnecessary courses [30]. This highlights the need for exploring new strategies to minimize patients’ exposure to unnecessary antibiotic courses. Convincing doctors, especially GPs, not to prescribe antibiotics when uncertain or under patient pressure is challenging. Despite the widespread practice, standard durations for common infections lack support from clinical studies [7]. However, recent randomized clinical trials, conducted mainly in the last 2 decades, show that ending antibiotic courses by day 2-3 for most RTIs is effective and safe, similar to early studies on short-term antibiotic courses for uncomplicated urinary tract infections carried out 20 years ago [47]. We believe health care professionals should encourage patients to stop antibiotic treatment promptly after symptom resolution unless a serious infection is suspected. In our clinical trial, these potential serious infections are clearly excluded. Continuing antibiotics beyond symptom resolution does not appear to prevent or reduce AMR. Conversely, reducing the duration of courses has been shown to curb the spread of AMR in pneumonia. It is essential to move away from strict, predetermined durations and adopt a patient-centered approach by customizing antibiotic courses to meet each patient’s individual needs. The key challenge involves identifying when exactly antibiotics can be safely stopped based on symptoms to minimize the chances of relapse. We currently lack evidence on how pathogen burden is associated with symptoms experienced by patients with RTIs.

STORM’s main focus on primary care settings makes it directly applicable to the daily practice of GPs who frequently encounter patients with uncomplicated RTIs. Infectious diseases represent one-third of all GP consultations, with over half of these visits attributed to RTIs [48]. This indicates that RTIs are among the most prevalent conditions encountered by GPs in their clinical practice. It acknowledges the challenges faced by health care professionals, particularly GPs, in resisting patient pressure and uncertainty when deciding whether to prescribe antibiotics. By advocating for a patient-centered approach to antibiotic prescribing, the proposal provides practical guidance for health care providers to tailor treatment regimens according to individual patient needs and clinical circumstances [49]. STORM draws upon recent clinical trials conducted over the past 2 decades, suggesting that the findings are based on contemporary evidence and thus likely applicable to current clinical practice.

To the best of our knowledge, there has not been any study in primary care exploring the feasibility and efficacy of stopping the antibiotic course once the clinical signs of the patient have improved and the CRP level is low. Furthermore, a randomized clinical trial has never been conducted to assess the effectiveness and safety of this crucial strategy in combating AMR and drug-related adverse events. Primary health centers are the first contact point for patients with RTI symptoms, which is why we will conduct the study in this setting. This will constitute the first clinical trial aimed at answering whether it is safe to shorten and tailor the antibiotic therapy duration to the needs of the patients. This study does not pursue the medicalization of tailoring the antibiotic course by requiring patients assigned to the intervention group to return for a clinical reassessment and CRP testing; however, this is the first trial of its kind. We aim to determine the efficacy and safety of this strategy. If tailoring antibiotic courses for RTIs proves to be as effective and safe as completing the entire course, further studies will investigate the necessity of the reassessment visit.

### Limitations

This is a pragmatic clinical trial evaluating the efficacy of different antibiotic therapy durations for acute RTIs, and therefore, masking techniques are not used. One limitation of this study is that a percentage of self-limiting viral infections will be included. While antibiotics are not the recommended treatment, they are still frequently prescribed for most RTIs worldwide [3]. However, all participants will receive a summary of the updated current guidelines, which discourage the use of antibiotics for most patients with acute rhinosinusitis, uncomplicated acute bronchitis, and acute COPD exacerbations without purulent sputum, leaving the decision to the clinician in this pragmatic approach. Nevertheless, we believe that the percentage of “viral self-limiting infections” included in this randomized clinical trial will be much lower than what is typically observed under normal conditions, as GPs will be informed about the appropriate indications for therapy and will have this information available before deciding whether to prescribe a β-lactam to the participant.

Both the primary and some of the secondary objectives of the study are based on information provided by the patients themselves. Regarding the CRP rapid testing, we are aware that high CRP levels can signal systemic inflammation in conditions like inflammatory disorders, not just infections. There is limited research on how CRP changes day by day in RTIs. Although levels above 100 mg/L might reflect the natural progression, we set conservative thresholds for this initial trial on shortening treatments as patients improve. Some centers are only open on weekdays, from Monday to Friday. In these cases, if a patient feels better over the weekend, they will need to wait until the following Monday to be assessed. To minimize this limitation, we will recruit only centers that remain open on Saturdays or, preferably, those that provide out-of-hours services. This strategy aims to ensure timely follow-up and reduce delays in clinical assessment.

The concept of “feeling better” is inherently subjective and can vary significantly across individuals, types of infections, and symptom profiles [50]. For some patients, it may indicate partial symptom improvement, while for others it may imply complete resolution of all symptoms. Certain symptoms, such as fever, might be a more reliable indicator for clinical improvement and the safe discontinuation of antibiotics, whereas symptoms like cough may persist longer without significantly impairing daily functioning. Additionally, some patients with RTIs may prefer to stop antibiotics only after full symptom resolution, while others may find the notion of symptom improvement too ambiguous, leading to uncertainty about when to discontinue treatment. To address this challenge, our study defines the appropriate time to stop antibiotic treatment based on a combination of clinical and patient-reported criteria: overall symptom improvement as reported by the patient, normalization of key vital signs—particularly body temperature, and the absence of elevated inflammatory markers, specifically CRP. As a pragmatic randomized clinical trial, this study is designed to provide real-world evidence on how patients interpret and respond to the concept of “feeling better” in the context of antibiotic use. Furthermore, to enhance our understanding, we will conduct a qualitative study with a subset of patients in the intervention group to explore their perceptions and experiences related to symptom improvement and treatment decisions. Insights from this qualitative component will help refine a more practical and clinically applicable definition of when it is appropriate to discontinue antibiotic therapy, ultimately supporting clinicians in providing more precise and consistent advice.

As with any randomized clinical trial, the STORM study faces some challenges. One potential difficulty is recruiting enough patients with acute RTIs who are willing to participate. Despite this moderate risk, the study is planned to run from October 2025 to April 2027, covering 2 winter periods, with the last patient recruited by March 2027. To ensure recruitment success, tight follow-up processes and various strategies will be implemented. These strategies may include inviting other professionals from existing centers to participate, extending the recruitment period by an additional 2 months if needed, and, if necessary, opening recruitment at additional sites to increase the pool of candidates. In addition, to minimize inclusion bias, consecutive recruitment of patients who meet the inclusion criteria and do not meet any exclusion criteria will be encouraged [51].

Professionals will follow up with all patients assigned to the intervention group on day 3 for assessment of the clinical condition and as a reminder to reach out to the center as they feel better. Professionals will be trained on how to explain the study’s objectives and the importance of adhering to the study procedures. For patients who do not return their symptom diaries, a short form will be completed via a phone call on day 14, asking about the number of days they took antibiotics, other therapies, and details regarding the duration and severity of their symptoms.

## Abbreviations

AMR: antimicrobial resistance
COPD: chronic obstructive pulmonary disease
CRP: C-reactive protein
e-CRF: electronic case report form
FAIR: Findable, Accessible, Interoperable, and Reusable
GP: general practitioner
QALY: quality-adjusted life year
REDCap: research electronic data capture
RTI: respiratory tract infection
SPIRIT: Standard Protocol Items: Recommendations for Interventional Trials
t.i.d.: 3 times daily

## Data Availability

The datasets generated or analyzed during this study are available from the corresponding author on reasonable request.
